# Comparison of the Force Exerted by Hippocampal and DRG Growth Cones

**DOI:** 10.1371/journal.pone.0073025

**Published:** 2013-08-21

**Authors:** Ladan Amin, Erika Ercolini, Jelena Ban, Vincent Torre

**Affiliations:** 1 Neuroscience Area, International School for Advanced Studies (SISSA), Trieste, Italy; 2 Cluster in Biomedicine (CBM), Area Science Park Basovizza, Trieste, Italy; 3 Italian Institute of Technology (IIT), Trieste, Italy; University of Iowa, United States of America

## Abstract

Mechanical properties such as force generation are fundamental for neuronal motility, development and regeneration. We used optical tweezers to compare the force exerted by growth cones (GCs) of neurons from the Peripheral Nervous System (PNS), such as Dorsal Root Ganglia (DRG) neurons, and from the Central Nervous System (CNS) such as hippocampal neurons. Developing GCs from dissociated DRG and hippocampal neurons were obtained from P1-P2 and P10-P12 rats. Comparing their morphology, we observed that the area of GCs of hippocampal neurons was 8-10 µm^2^ and did not vary between P1-P2 and P10-P12 rats, but GCs of DRG neurons were larger and their area increased from P1-P2 to P10-P12 by 2-4 times. The force exerted by DRG filopodia was in the order of 1-2 pN and never exceeded 5 pN, while hippocampal filopodia exerted a larger force, often in the order of 5 pN. Hippocampal and DRG lamellipodia exerted lateral forces up to 20 pN, but lamellipodia of DRG neurons could exert a vertical force larger than that of hippocampal neurons. Force-velocity relationships (*Fv*) in both types of neurons had the same qualitative behaviour, consistent with a common autocatalytic model of force generation. These results indicate that molecular mechanisms of force generation of GC from CNS and PNS neurons are similar but the amplitude of generated force is influenced by their cytoskeletal properties.

## Introduction

Neuronal motility is at the basis of several major functions, such as neuronal development, memory, repair and cell migration [1]. During the accomplishment of these functions, neurons protrude neurites, highly motile structures which explore the environment searching for the appropriate chemical or mechanical cues guiding the formation of correct connections [2]. Neurite exploration is guided by growth cones (GCs) located at their tip [3–5], formed by an extended lamellipodium from which thin filopodia emerge [6]. Filopodia tips can move at a velocity that can reach 0.8-1 µm/s [7–9] and their motility is at the basis of the efficient formation of neural networks.

The primary source of motility in GCs is the polymerization of actin filaments [8,9], a process controlled by a variety of regulatory proteins [10]. The addition of actin polymers to actin filaments in close contact with the membrane pushes the cellular membrane forward exerting a protrusive force [11,12]. Mathematical modeling provides a way to link known molecular events to force generation processes. A key outcome of these models is represented by the force – velocity (*Fv*) relationships, describing how the force (*F*) exerted by the actin filament network is related to the velocity (v) of their growing ends. Different shape of *Fv* relationships are expected from autocatalytic models [13,14] or Ratchet models [9,15]. Several other mathematical models have been developed [16] providing a link between measured forces and underlying molecular events. By using optical tweezers [17,18], we previously measured the force exerted by lamellipodia and filopodia from developing GCs of isolated Dorsal Root Ganglia neurons (DRG) [19]. The force exerted by filopodia was in the order of 1-2 pN and never exceeded 5 pN, while lamellipodia exerted large forces up to 20 pN.

This quantitative characterization of force generation was carried out in DRG neurons i.e sensory neurons of peripheral nervous system (PNS) which are characterized by GCs with an extended lamellipodium [20].

Therefore we asked whether the morphology and the force exerted by hippocampal neurons, belonging to the central nervous system (CNS) were different. In addition, DRG neurons that we previously investigated were obtained from P10-P12 rats and it is possible that neurons isolated from rats at different developmental stages exert a force with a different strength.

DRG neurons can be prepared from embryonic, postnatal, or adult tissue. Embryonic tissue contains few glial cells and has the advantage of providing a higher cell yields with a greater proportion of neurons but they depend on the neurotrophins for survival [21]. In contrast, postnatal and adult DRG sensory neurons are a good model of mature and completely developed neurons.

The aim of the present investigation is to measure and compare the force exerted by filopodia and lamellipodia of both CNS and PNS neurons at different developmental stages. Therefore we compared force generation in DRG and hippocampal neurons obtained from P1-P2 and from P10-P12 rats. Our results suggest that molecular mechanisms of force generation is similar in different neurons, but the specific organization of the cytoskeleton inside filopodia and lamellipodia influence and determine the amplitude of generated force.

## Materials and Methods

### Cell culture preparation

P1-P2 and P10-P12 Wistar rats were sacrificed by decapitation after being anesthetized by CO2 in accordance with the guidelines of the Italian Animal Welfare Act and their use has been approved by the Local Veterinary Service, by SISSA Ethics Committee board and by National Ministry of Health (Permit Number: 2189-II/7), as they are in accordance with the European Union guidelines for animal care (d.1.116/92; 86/609/C.E.). All efforts were made to minimize suffering. We confirm that the Ethics Committee of the International School for Advanced Studies (SISSA) has approved the protocol (Permit Number: 2189-II/7).

#### DRG neurons

DRG neurons were obtained as previously described [19,22,23]. Briefly, Wistar rats 1-2 days old (P1-P2) and 10 to 12 days old (P10-P12) were sacrificed by decapitation after being anesthetized by CO_2_. After dissection, DRGs were incubated with 0.5 mg/ml trypsin, 1 mg/ml collagenase and 0.1 mg/ml DNase (all from Sigma-Aldrich, St. Louis, MO) in 5 ml Neurobasal medium (Life Technologies, Gaithersburg, MD) in a shaking bath (37°C, 40 minutes). DRGs were mechanically dissociated, centrifuged at 300 rpm, resuspended in medium and plated on 0.5 mg/ml poly-L-lysine (Sigma-Aldrich, St. Louis, MO) coated coverslips at low density. DRG neurons were incubated for 24 h to 48 h and Nerve growth factor (50 ng/ml; Alomone Labs, Jerusalem, Israel) was added before performing the measurements.

#### Hippocampal neurons

After decapitation, hippocampi of P1-P2 or P10-P12 rats were dissected, cut into slices and washed twice with the dissection medium [24]. The enzymatic dissociation was performed treating the slices with 5 mg/ml trypsin (Sigma-Aldrich, St. Louis, MO) and 0.75 mg/ml DNase I (Sigma-Aldrich, St. Louis, MO) in digestion medium (5 min, room temperature). Then, trypsin was neutralized by 1 mg/ml trypsin inhibitor (Sigma-Aldrich, St. Louis, MO) in the dissection medium for 10 minutes on ice. After wash in the dissection medium, mechanical dissociation was performed in the same dissection medium with 0.6 mg/ml DNase I by approximately 50 passages through a Gilson P1000 tip. The cell suspension was then centrifuged at 800 rpm for 5 min, and the pellet re-suspended in the culture medium. Finally, hippocampal neurons were plated on 50 µg/ml poly-L-ornithine (Sigma-Aldrich, St. Louis, MO) coated coverslips. The hippocampal neuronal cultures were incubated (5% CO_2_, 37^°^C) for 24-48 hours in the minimum essential medium with Earle’s salts and Glutamax I with 10% FBS, 2.5 µg/ml gentamycin (all from Invitrogen, Life Technologies, Gaithersburg, MD, USA), 6 mg/ml D-glucose, 3.6 mg/ml Hepes, 0.1 mg/ml apo-transferrin, 30 µg/ml insulin, 0.1 µg/ml biotin, 1.5 µg/ml vitamin B12 (all from Sigma-Aldrich, St. Louis, MO).

The number of glial cells in rat brain markedly increases during the weeks after birth therefore in postnatal cultures sometimes glial cell overgrowth represent a problem. The overall goal of our dissection protocols were to culture highly purified hippocampal or DRG neurons by carefully isolating the tissue, plating at a low cellular density and culture neurons for 24-48 h. To arrest glial cell proliferation, on the second day we treated the culture with 5 µM cytosine**-**β*-*D**-**arabinofuranoside ((Ara-C), Sigma-Aldrich, St. Louis, MO). Isolated GCs from identified neurons were chosen for force measurements.

### Optical tweezers setup

The optical tweezers setup was built as described in [25] and it is extensively described in [19,22]. The trapping source was an Ytterbium fiber laser operating at 1064 nm (IPG Laser GmbH, Burbach, Germany) which was sent onto an inverted microscope (IX81, Olympus, Milan, Italy) to the focusing objective (Olympus 100X oil, NA 1.4). The dish containing the differentiating neurons and the beads (PSI-1.0NH2, G. Kisker GbR, Steinfurt, Germany) was placed on the microscope stage and its temperature was kept at 37°C by a Peltier device. The dish was maintained in an environment without a controlled level of CO_2_. The bead position **x** = (*x*, *y*, *z*) was determined with a lateral and vertical accuracy of 2 and 5 nm, respectively, by using back focal plane detection [17,26]. The trap stiffness K_x,y,z_ = (*k*_*x*_, *k*_*y*_, *k*_*z*_) and the detector sensitivity were calibrated using the power spectrum method [17]. The force exerted by the lamellipodium or by the filopodium **F** was considered equal to **-F_trap_**. When the displacement of the bead from its equilibrium position inside the trap **d** = (*d*_*x*_, *d*_*y*_, *d*_*z*_) was less than 400 nm and 250 nm vertically and laterally, respectively, **F_trap_** = (*F*_*x*_, *F*_*y*_, *F*_*z*_) was calculated as *F*_*x*_ = *k*_*x*_ *d*_*x*_, *F*_*y*_ = *k*_*y*_ *d*_*y*_, and *F*_*z*_ = *k*_*z*_ *d*_*z*_ [17]. All individual force recording experiments which lasted for 150 s were monitored by video imaging with a CCD camera at a frame rate of 5 Hz. The determination of the linear range and the sensitivity of the optical trap are described in detail in [22].

### Computation of Fv relationships

Details of the computation of *Fv* relationships as well as of the determination of the bandwidth of biological events underlying force generation can be found in [19]. Briefly, the velocity v = (*v*_*x*_, *v*_*y*_, *v*_*z*_) of the bead was obtained by numerical differentiation of its sampled position x = (*x*(*n*), *y*(*n*), *z*(*n*)) *n* = 1… N. Numerical differentiation was computed either by convolution of the position components *x*(*n*), *y*(*n*) and *z*(*n*) with the derivative of a Gaussian filter 1/[ó(2ð)^^1 /2^^] exp(-*t* ^2^/ ó^2^) (Gaussian filtering) or by Linear regression. Gaussian filters corresponding to cut-off frequencies of 0.2, 1 and 10 Hz were used.

### Immunostaining and imaging

Cells were fixed in 4% paraformaldehyde containing 0.15% picric acid in phosphate-buffered saline (PBS), saturated with 0.1 M glycine, permeabilized with 0.1% Triton X-100, saturated with 0.5% BSA (all from Sigma-Aldrich, St. Louis, MO) in PBS and then incubated for 1hour with TUJ1, mouse monoclonal antibody against neuronal class III β-tubulin (Covance, Berkeley, CA) followed by the 30 min incubation with goat anti-mouse 594 *Alexa* and *Alexa* Fluor 488 phalloidin (all from Invitrogen, Life Technologies, Gaithersburg, MD, USA). All the incubations were performed at room temperature (20-22^°^C). Since microtubules are sensitive to fixation procedure several fixation protocols was tested (more details can be found in Information S1). The fixation protocol with paraformaldeyde provided best staining of both actin and microtubules. Cells were examined using a Leica DMIRE2 confocal microscope (Leica Microsystems GmbH, Germany) equipped with DIC and fluorescence optics, Ar/ArKr 488nm and He/Ne 543/594nm lasers. The fluorescence images (1024x1024 pixels) were collected with a 63X magnification and 1.4 NA oil-immersion objective. For the neurite’s length analysis Leica DM6000 microscope equipped with CCD camera, DIC and fluorescence optics was used. Images were acquired with 40X magnification (1.0 or 1.3 NA) oil-immersion objectives.

### Neurite and filopodia length and lamellipodia area measurement

The length of the neurites and filopodia was measured from confocal images of actin stainings using the software: NeuriteTracer (ImageJ plugin) [27], (http://dx.doi.org/10.1016/j.jneumeth.2007.08.029).

### Statistical analysis

Data are shown as means ± standard error (SE). Statistical significance was evaluated using the t-student test. A p value of 0.05 was considered to be statistically significant.

## Results

DRG and hippocampal neurons of P1-P2 and of P10-P12 Wistar rats were isolated and plated on poly-L-lysine-coated and poly-L-ornithine coated coverslips respectively. After 24-48 h of culture, coverslips containing either DRG or hippocampal neurons were positioned on the stage of an inverted microscope used for imaging and force measurement [19] (see also Materials and Methods). Silica beads with a diameter of 1 µm were trapped with an infrared (IR) optical tweezer in front of GCs and it was possible to measure the force exerted by neuronal filopodia and lamellipodia with sub pN sensitivity at 10 kHz resolution.

### Force-velocity relationships from hippocampal and DRG lamellipodia

The force necessary for GC motility which cause the neurite to explore the environment, grow, retract, turn and branch is generated by the combination of actin and MTs dynamics coupled with myosin-based retrograde actin flow and the selective adhesion to extracellular substrate [28–30].

In order to verify and to compare the molecular mechanism underlying the force generation in CNS and PNS neurons, we determined *Fv* relationships with millisecond (ms) temporal resolution and picoNewton (pN) sensitivity so to verify whether the same mechanism governed force generation in both type of neurons.

**Figure 1:**
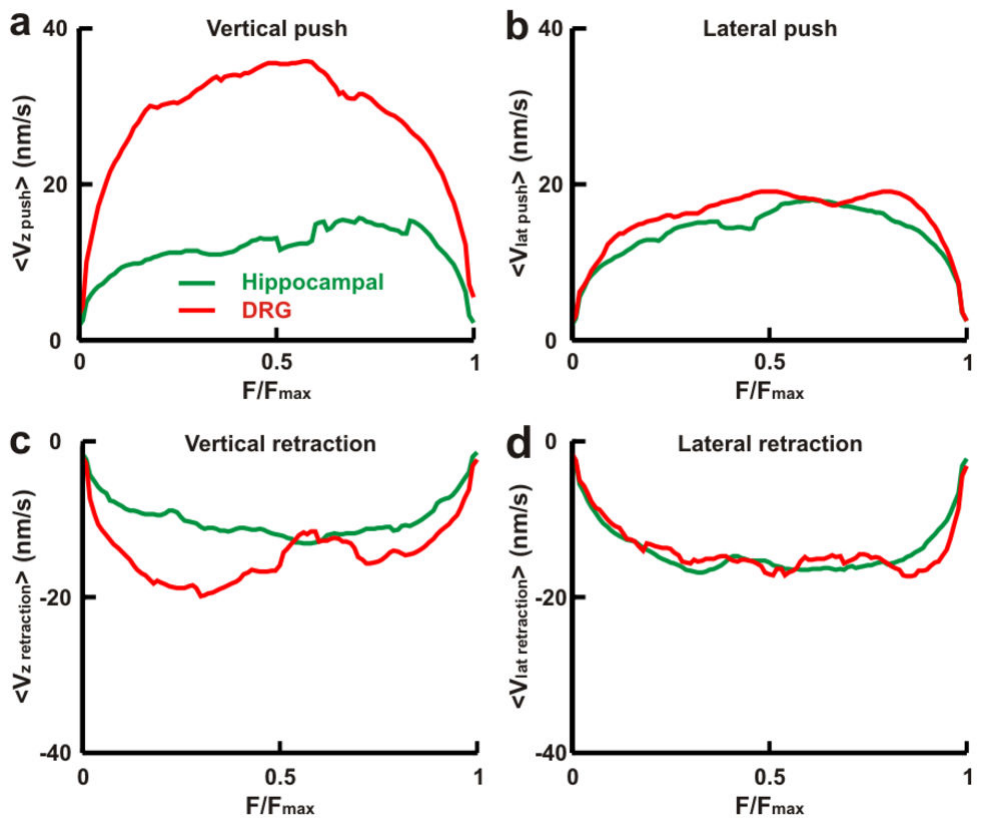
*Fv* relationships during pushes and retractions from hippocampal and DRG lamellipodia. (*a*–*d*) Average *Fv* relationships, <*Fv*>_0.2_, normalized to *F*_*max*_ for vertical pushes (*a*), lateral pushes (*b*), vertical retractions (*c*) and lateral retractions (*d*) for hippocampal and DRG lamellipodia. The numbers of individual *Fv* relationships averaged in DRG and in hippocampal neurons were equal to (*a*) 23, 14, respectively; (*b*) 20, 34, respectively; (*c*) 23, 14, respectively; and (*d*) 14, 14, respectively. DRG data are taken from our previous work [23].

We computed average *Fv* relationships, <*Fv*>, from the measured displacements and forces for vertical and lateral pushes and retractions [19,22] both for hippocampal and DRG lamellipodia from P10-P12 rats. Vertical refers to the direction perpendicular to the coverslip (*z* axis) and lateral refers to the plane of the coverslip (*x*, *y*). *Fv* relationships obtained from a single experiment (see Materials and Methods and [19] were normalized to *F*_*max*_ and were averaged so to obtain average *Fv* relationships, <*Fv*>. At the beginning, the bead is in the trap far from the lamellipodia and its velocity is zero. During a push, the lamellipodia leading edge moves toward the trapped bead with constant velocity [14]. Before reaching a solid contact with the bead, the bead’s velocity increases, but later on, after the contact has been made and is complete, bead and lamellipodia move with the same constant velocity. Therefore, during vertical pushes <*Fv* > was characterized by an initial rise of *v* reaching the value of ~ 35 nm/s for DRG lamellipodia and ~ 15 nm/s for hippocampal lamellipodia (Figure 1 a). <*Fv* > relationships during lateral pushes (Figure 1 b) and retractions (Figure 1 d) were very similar for hippocampal and DRG lamellipodia. For vertical retractions (Figure 1 c) the shape of <*Fv* > relationships was very similar but had a higher velocity for DRG lamellipodia - up to 19 nm/s - while in hippocampal lamellipodia it was not higher than 12 nm/s. Therefore the same molecular mechanism – possibly the autocatalytic model [13,14] - seems to control force generation in both kind of neurons, but the amplitude of developed force and velocity depends of the specific kind of neurons.

### Force measurements in hippocampal and DRG filopodia and lamellipodia

**Figure 2:**
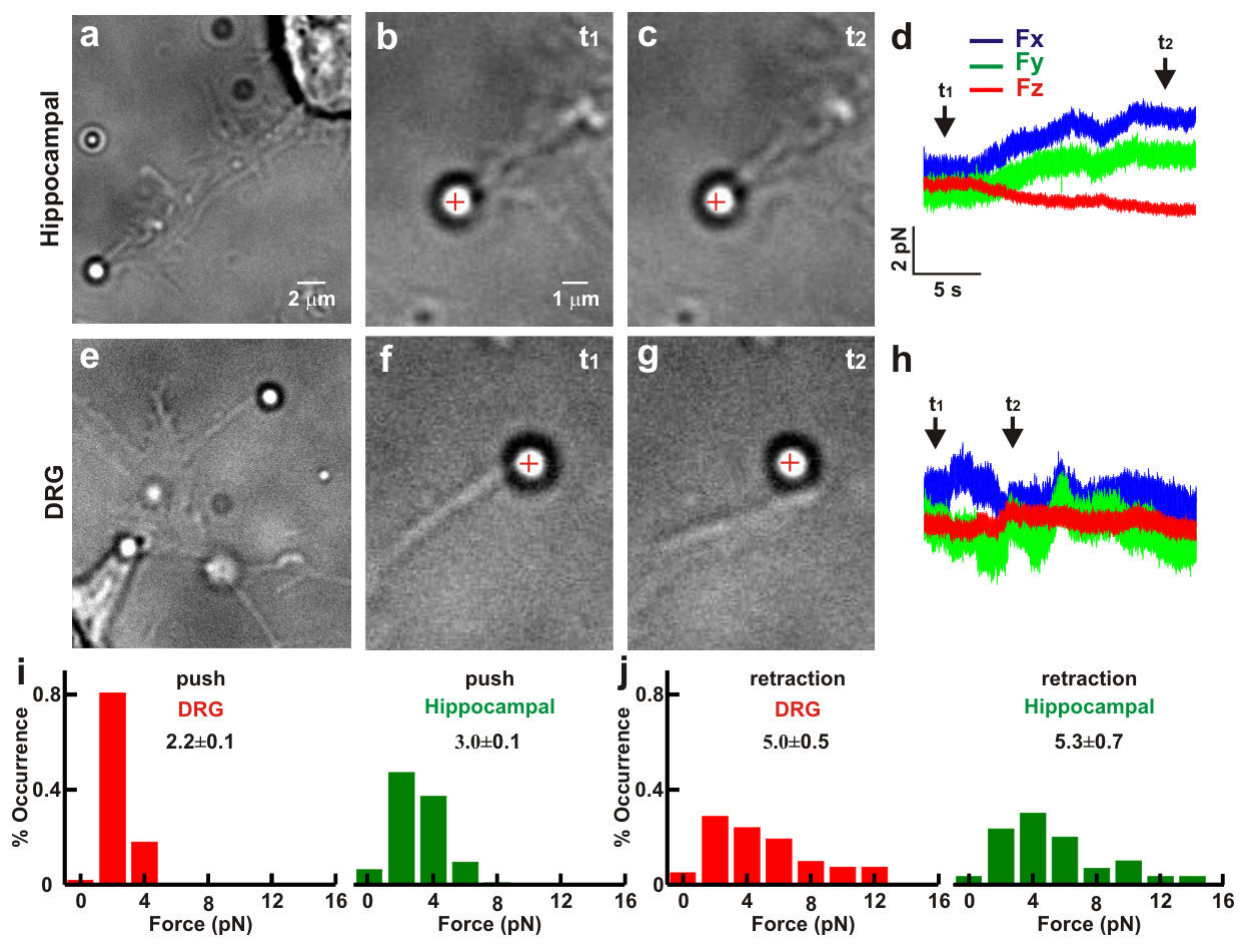
Comparison of the force exerted by filopodia from hippocampal and DRG GCs. (*a*) Low resolution image of a bead trapped in front of a filopodium emerging from a GC of hippocampal neuron. (*b*–*c*) High resolution images during a push by a filopodium. At *t*_*1*_ the bead is in the optical trap (*b*) and at *t*_*2*_ the filopodium pushes the bead (*c*). The cross indicates the center of the optical trap. (*d*) The three components *F*_*x*_, *F*_*y*_, and *F*_*z*_ of the force exerted by the filopodium. (*e*–*h*) As in (*a*–*d*) for a filopodium emerging from a GC of DRG neuron. (*i*) Histogram of the force measured during a push in DRG (n=58) and hippocampal (n=64) neurons. (*j*) As in (*i*) but during retraction (n=31 and n=23 respectively). The trap stiffness is *k*_*x,y*_=0.10, *k*_*z*_=0.03 pN/nm. All values reported in *i-j* are given as mean ± SE.

Filopodia of hippocampal and DRG GCs have a similar elongated shape with a diameter varying from 80 to 400 nm [31] and an average length of 3.6±0.2 and 5.9±0.6 µm, respectively. In order to measure the force they exert, we positioned a silica bead trapped with an IR laser beam in front of filopodia tips (Figure 2 a and e). Protruding filopodia pushed trapped beads and displaced them from their equilibrium position inside the optical trap both for P1-P2 hippocampal (Figure 2 b and c) and P10-P12 DRG filopodia (Figure 2 f and g). During these protrusions, filopodia exerted a lateral force up to 2-4 pN and often also along the vertical axis but rarely exceeding 2 pN (Figure 2 d and h). Collected data indicate that DRG filopodia during protrusions exerted an average force of 2.2 ±0.1 pN (n=58) lower than the force exerted by hippocampal filopodia equal to 3.0 ±0.1 pN (n=64) (Figure 2 i). Often filopodia could seal on the silica bead, so that when they retracted they pulled the bead away from the optical trap, exerting a force during their retraction. During retractions both DRG and hippocampal filopodia exerted a force significantly larger than during protrusion, equal to 5.0±0.5 (n=31) and 5.3±0.7 pN (n=23), respectively (Figure 2 j).

**Figure 3:**
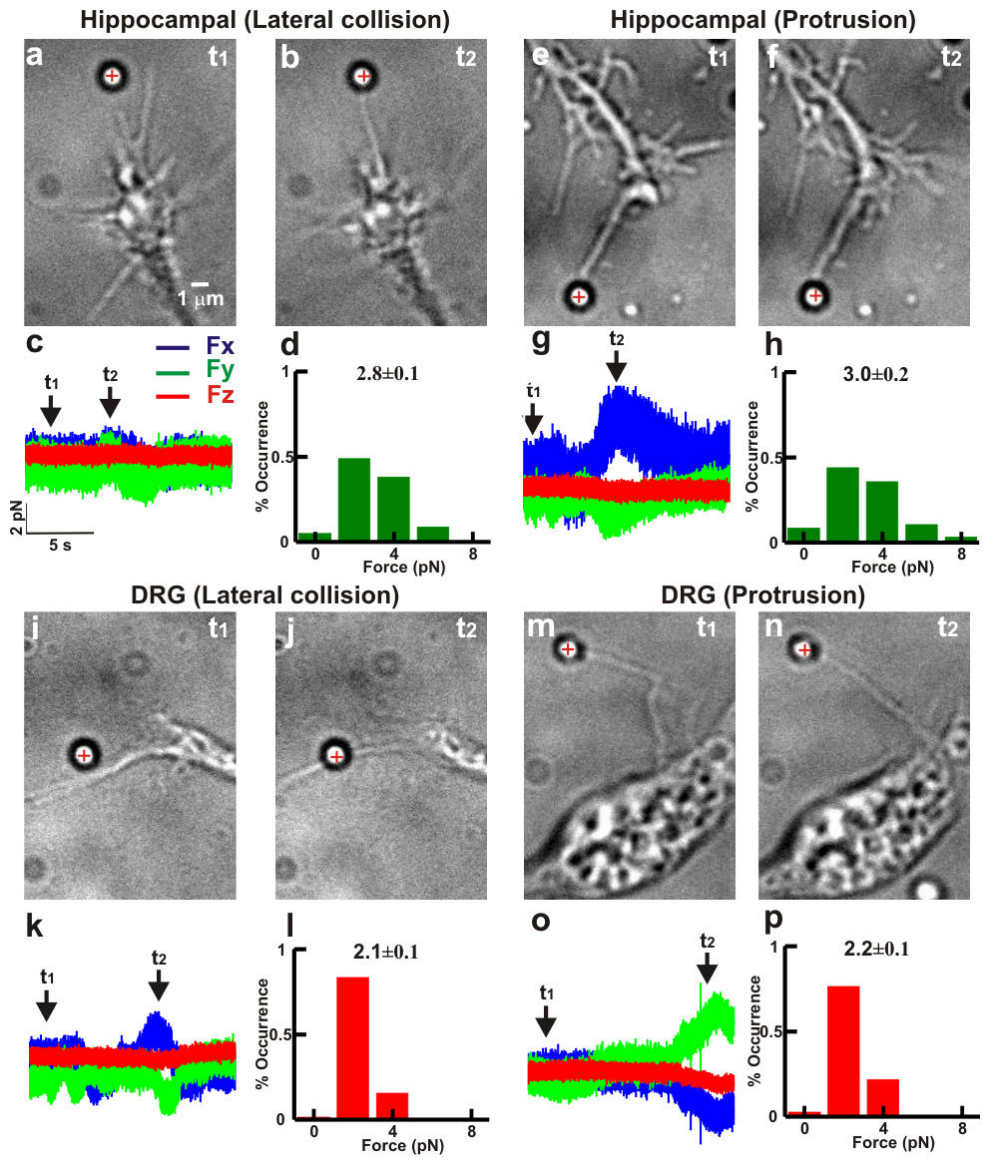
Lateral collision and protrusion of filopodia. (*a*–*b*) Lateral collision between a filopodium from hippocampal neuron and a trapped bead. The cross indicates the bead’s equilibrium position inside the optical trap. (*c*) *F*_*x*_, *F*_*y*_, and *F*_*z*_ during the lateral collision shown in (*a*–*b*). (*d*) Histogram of force measured during lateral collision in hippocampal neurons (n=41). (*e*–*f*) Collision between a protruding filopodium from hippocampal neuron and a trapped bead. (*g*) *F*_*x*_, *F*_*y*_, and *F*_*z*_ during the filopodial protrusion shown in (e–*f*). (*h*) Histogram of forces measured during protrusions (n=24). (*i*–*l*) As in (*a*–*d*) for a filopodium from a DRG neuron (n=39). (*m*–*p*) As in (*e*–*h*) for a protruding filopodium from a DRG neuron (n=22). All values reported in *d, h, l* and *p* are given as mean ± SE.

During their exploratory motion often filopodia pivot and push beads aside, possibly as a consequence of shearing movements of the lamellipodial actin network where the filopodial shaft emerges. We refer to the first case as lateral collisions (Figure 3 *a-b* and *i-j*) and to the latter case, where the filopodium pushes the bead, as protrusion (Figure 3 *e-f* and *m-n*). The amplitude of exerted force and their time course was similar for DRG and hippocampal filopodia both for lateral collisions (Figure 3 c and k) and protrusions (Figure 3 g and o). The force exerted during lateral collisions depends on the geometry of the collision, since a filopodium, during its exploratory motion, can hit the bead by slightly touching it with its tip (as in Figure 3 b) or hitting it with an intermediate part of the shaft (as in Figure 3 j). Histograms of the force measured during lateral collisions are shown in Figure 3 d and l, and during protrusions in Figure 3 h and p (hippocampal and DRG filopodia, respectively).

Simple mechanical considerations show that the force exerted by a wandering filopodium during a lateral collision [25] can be accounted for by the elastic force expected from its flexural rigidity [11,32] and its bending or buckling. No additional contribution from other force-generating mechanisms is required.

**Figure 4:**
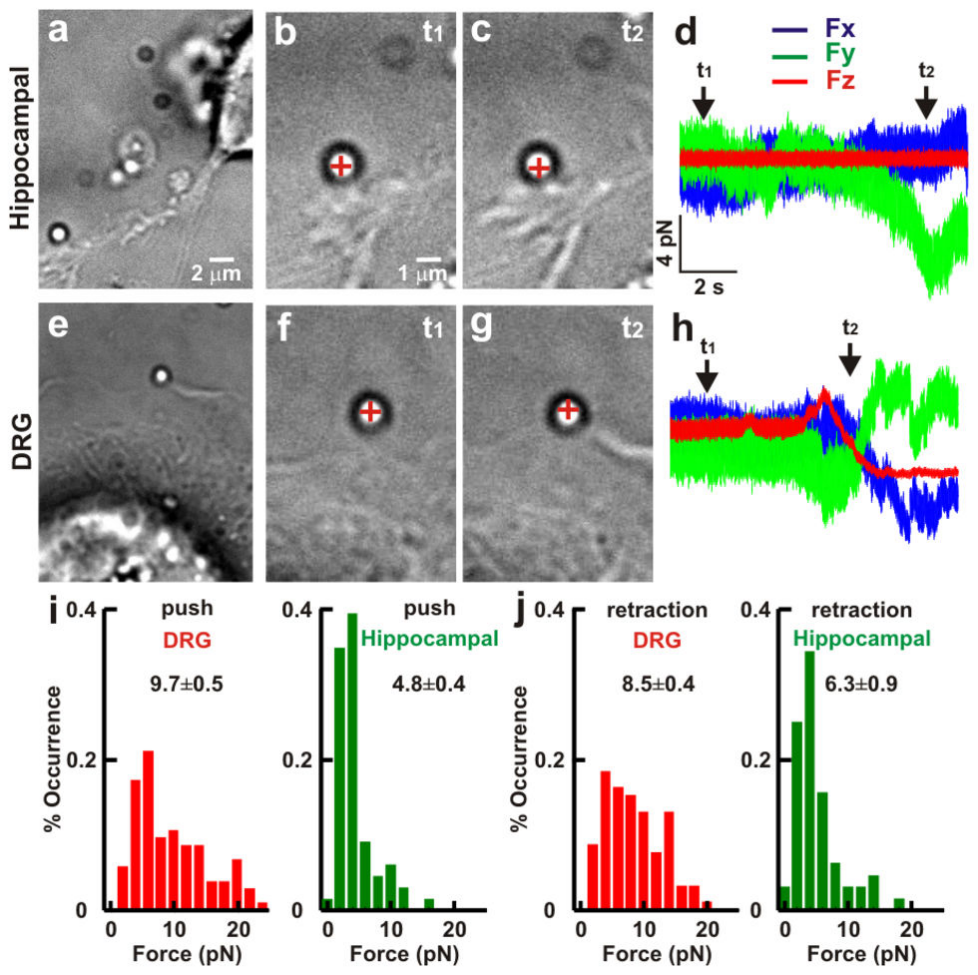
Comparison of the force exerted by lamellipodia from hippocampal and DRG GCs. (*a*) Low resolution image of a bead trapped in front of a lamellipodium emerging from a hippocampal neuron. (*b*–*c*) High resolution images during a push by a lamellipodium. At *t*_*1*_ the bead is in the optical trap (*b*) and at *t*_*2*_ lamellipodium grows and pushes the trapped bead (*c*). The cross indicates the center of the optical trap. (*d*) The three components *F*_*x*_, *F*_*y*_, and *F*_*z*_ of the force exerted by the lamellipodium from hippocampal neuron. (*e*–*h*) As in (*a*–*d*) but for a lamellipodium emerging from a DRG neuron. (*i*) Histogram of force measured during push in DRG (n=51) and hippocampal neurons (n= 31). (*j*) As in (*i*) but during retraction (n=47 and n=17 respectively). The trap stiffness is *k*_*x,y*_=0.10, *k*_*z*_=0.03 pN/nm. All values reported in *i-j* are given as mean ± SE.

As in the case of filopodia, silica beads were trapped in front of lamellipodia (Figure 4 a) and when the lamellipodia grew, they displaced the bead (Figure 4 b and c) exerting a force up to 20 pN. Growing lamellipodia could displace beads exclusively in the lateral direction (Figure 4 b–d) but more often they displaced beads both laterally and vertically (Figure 4 f–h). Collected data show that P10-P12 DRG lamellipodia exerted an average force of 9.7 ±0.5 (n=51) significantly larger than the average force of 4.8 ±0.4 pN (n=33) exerted by P1-P2 hippocampal lamellipodia (Figure 4 i).

When the lamellipodium retracted and the bead was attached to its membrane, the force exerted by a lamellipodium during the retraction could be measured (Figure 4 e–g). Force recordings (Figure 4 h) show that the lamellipodium retracted both in the lateral and vertical directions. Very often, the adhesion of the bead to the lamellipodial membrane was so strong that the bead did not jump back into the trap and remained tightly attached to the GC.

In DRG lamellipodia, forces during vertical pushes are larger than in hippocampal lamellipodia with mean values of 3.9±0.3 pN and 1.0±0.2 pN, respectively (Table 1). Measured forces during lamellipodia retraction had larger values in DRG lamellipodia and were 8.5±0.4 pN and 6.3±0.9 pN (Figure 4 j) for DRG and hippocampal lamellipodia respectively (see Table 1 for details). During vertical retraction, measured forces reached values up to 10 pN in DRG lamellipodia but in hippocampal lamellipodia the maximum value was very rarely larger than 4 pN with mean values of 4.1±0.3 and 2.0±0.3 pN, respectively (Table 1). No significant difference in the force exerted by filopodia or lamellipodia of P1-P2 or P10-P12 neurons was seen in hippocampal neurons.

**Table 1:** Force generated by filopodia and lamellipodia of hippocampal and DRG neurons.

		**Hippocampal**		**DRG**	
		F_x,y_ (pN)	F_z_ (pN)	F_x,y_ (pN)	F_z_ (pN)
**filopodia**	push	3.0±0.1	0.7 ± 0.1	2.2±0.1	0.6±0.1
	retraction	5.3±0.7	1.5 ± 0.2	5.0±0.5	1.3±0.2
**lamellipodia**	push	4.8±0.4	1.0 ± 0.2	9.7±0.5	3.9±0.3
	retraction	6.3±0.9	2.0 ± 0.3	8.5±0.4	4.1±0.3

Values represent the mean ± SE.

**Figure 5:**
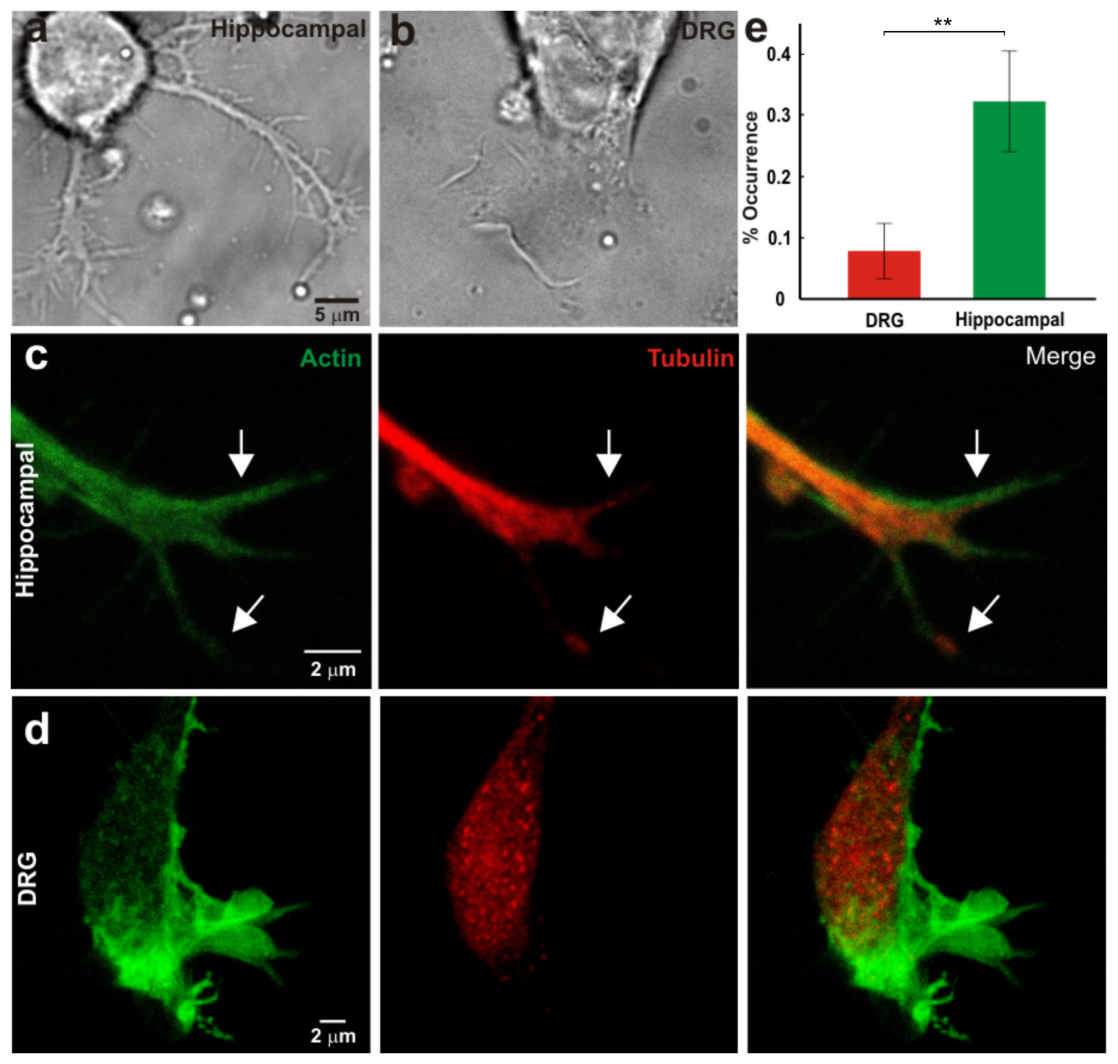
Morphoogical properties of hippocampal and DRG GCs. (*a*–*b*) Low resolution image of neurites emerging from the soma of hippocampal (*a*) and DRG (*b*) neuron. (*c*) From left to right: confocal fluorescence images of a hippocampal GC stained for actin, tubulin and merge of the two stainings. Arrows indicate a filopodium with MTs inside. (*d*) As in (*c*) but for DRG GC. (*e*) The fraction of filopodia with a staining for MTs in DRG and hippocampal GC. Error bars indicate the SE. Significance indicates p < 0.01 (Student’s t-test).

The results of Figures 2-4 and Table 1 indicate that hippocampal filopodia exert a larger force than DRG filopodia but DRG lamellipodia exert a larger vertical push. These differences are likely to be related to their different cytoskeleton organization (see Figure 5).

### Morphological properties of hippocampal and DRG GCs

The morphology of hippocampal and DRG neurons is rather different and when cultivated in a dish they can be easily recognized. After 6-12 hours of culture, as previously observed [33], thin neurites emerge from the soma of hippocampal neurons (Figure 5 a) while extended lamellipodia sprout from the soma of DRG neurons (Figure 5 b). Neurites emerging from hippocampal neurons can grow extensively up to some tens of µm and occasionally could retract. After 12 hours of culture, neurites start to emerge also from the soma of DRG neurons and follow dynamics similar to that observed in hippocampal neurons.

**Figure 6:**
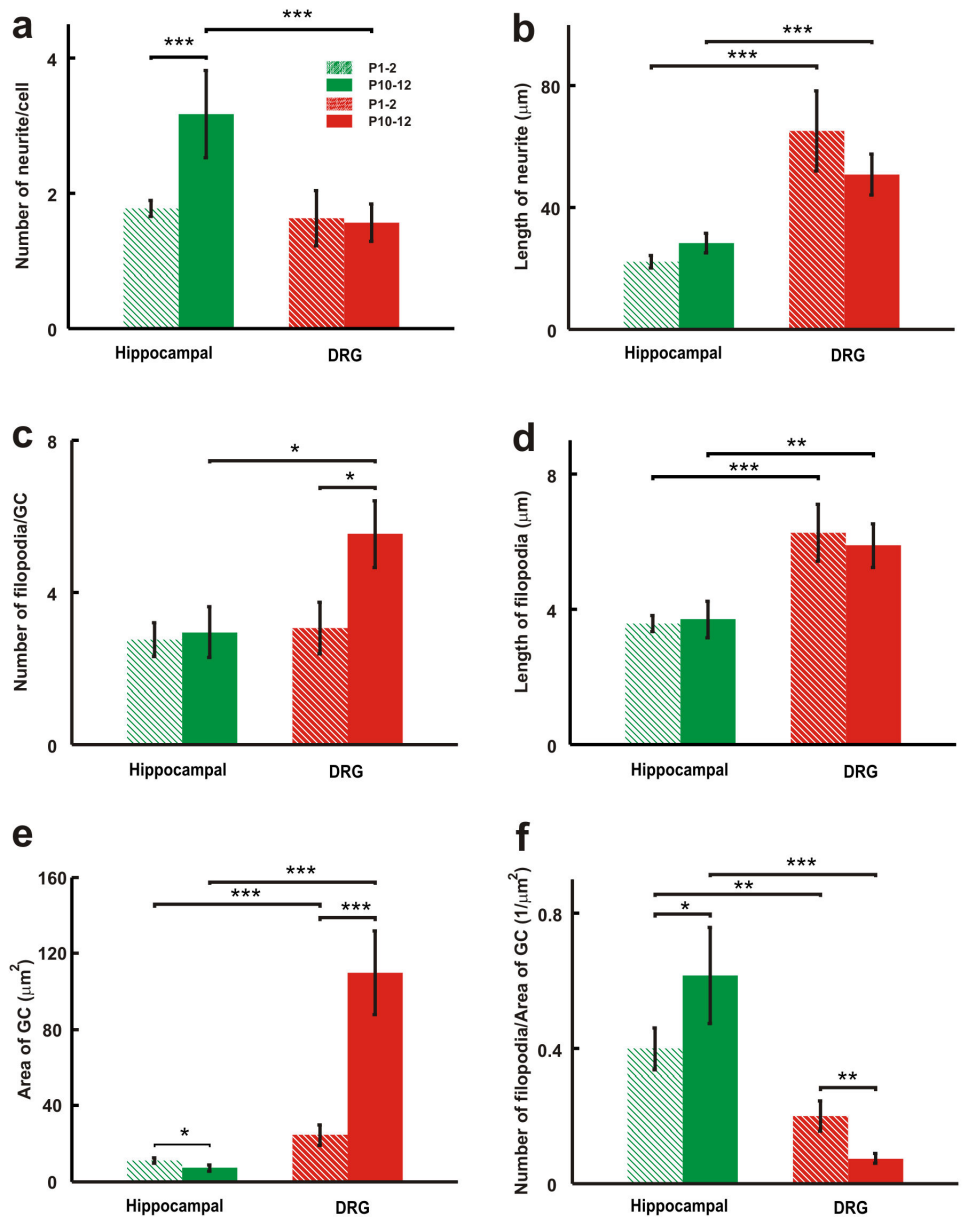
Differences in the morphology of DRG and hippocampal neurites and GCs. (*a*) Average number of neurites emerging from hippocampal and DRG soma. (*b*) Average neurite lengths from the tip of each neurite to the edge of the hippocampal and DRG soma. (*c*) Average number of filopodia emerging from hippocampal and DRG GCs. (*d*) Average filopodium lengths from the tip of each filopodia to the edge of the hippocampal and DRG GCs. (*e*) Average area of hippocampal and DRG GCs. (*f*) Ratio of number of filopodia and area of GC in hippocampal and DRG GCs. In all panels solid bars refer to P10-P12 neurons and striped bars indicate the P1-P2 neurons. Data are shown as mean ± SE. Significance indicates p < 0.05, p < 0.01 and p < 0.001 (*, **, *** respectively, Student’s t-test).

DRG and hippocampal cultures were fixed at 24 hours after plating, so to prevent neurites from forming a network. Morphological differences were analyzed by immunofluorescence with specific probes for F-actin (phalloidin) and MTs (β-tubulin-III antibody TUJ1). Quantitative details are summarized in Figure 6.

The number of neurites emerging from hippocampal and DRG neurons is different. In DRG cultures (P10-P12 and P1-P2) most of the observed neurons have 1 (63%), 2 (28%) or 3 (6%) neurites and only occasionally neurons show up to 6 (3%) neurites. Most of plated hippocampal neurons (P1-P2) generated either one or two neurites (44% and 40% respectively) and only 16% of these cells had three or more neurites. But the number of neurites generated from P10-P12 hippocampal neurons is significantly higher and almost 80% of these neurons have more than 3 neurites (Figure 6 a). Neurites from DRG neurons of both P1-P2 and P10-P12 rats have a length of 65.6±8.0 and 50.7±5.8 µm respectively and are longer than those from hippocampal neurons, whose length was 22.2±2.0 and 28.2±2.4 µm, respectively (Figure 6 b). GCs emerging from DRG and hippocampal neurons have different morphology and motility. Although the size of GCs varies significantly, the average size of a DRG GC is several times larger than the size of a hippocampal GC (Figure 6 e). In hippocampal GCs several filopodia emerging from a lamellipodium are significantly less extended than those from a DRG lamellipodium (Figure 5 c and d). In hippocampal neurons, the GC size is almost constant at different development stages (P1-P2 and P10-P12 rats). But in DRG neurons, P10-P12 GC lamellipodia are larger than those of P1-P2 GCs (Figure 6 e). The ratio between the number of filopodia and GC area is larger in hippocampal GCs (0.40±0.10 µm^-2^) than in DRG GCs (0.10± 0.02 µm^-2^) (Figure 6 f). Therefore, hippocampal neurons seem to be more “filopodial” while GCs have a more bundle-like structure. Hippocampal GCs have shorter filopodia and their length remains constant in both P1-P2 and P10-P12 GCs (Figure 6 d).

In summary, we have not observed any statistical significant difference in neurons dissociated from P1-P2 and from P10-P12 rats except for the number of neurites in hippocampal neurons and the size of GCs in DRG neurons which are higher in more mature animals. The time of culture seems to be the major determinant of neurites’ length and not the animal’s age.

GCs from DRG and hippocampal neurons, not only differ in their morphology, but also in the organization of their cytoskeleton. Immunostaining of GCs for actin and tubulin shows that in hippocampal GCs MTs extend into the periphery domain (P domain) and even penetrate inside filopodia (Figure 5 c). In DRG GCs MTs usually terminate at the central domain (C domain) and only rarely (less than 10%) protrude into the P domain (Figure 5 e).

Taken together data shown in Figures 5 and 6 indicate two main differences of the cytoskeletal organization of DRG and hippocampal GCs: firstly, MTs enter inside hippocampal filopodia more often than in DRG filopodia and, secondly, DRG lamellipodia are larger by 2-4 times than in hippocampal GCs.

## Discussion

The present manuscript shows that filopodia and lamellipodia from GCs of CNS - hippocampal neurons - and PNS-DRG neurons - are both able to exert forces. The analysis of *Fv* relationships in the two types of neurons suggests a similar molecular mechanisms underlying force generation in both types of neurons. Hippocampal filopodia, however, exert a force larger than DRG filopodia, while DRG lamellipodia exert a larger force during vertical pushes.

### Fv relationships

Experimental characterization of *Fv* relationships in DRG and hippocampal GCs shows that <*Fv* > relationships in both types of neurons exhibited a flat shape, during which the mean velocity remained constant while the force increased (Figure 1). <*Fv* > relationships during pushes and retractions (Figure 1) were very similar. Therefore, the autocatalytic model [13,14] correctly describes force generation in both types of neurons. During vertical pushes and retractions <*Fv* > relationships had a higher velocity for DRG than for hippocampal lamellipodia. *<Fv>* relationships describe force generation in a mean field approximation, suggesting common basic molecular mechanisms in both kind of neurons, with quantitative differences in the maximal amplitude of *F* and *v* likely caused by their different cytoskeletal organization (Figure 5).

### Hippocampal and DRG filopodia

A very important difference between hippocampal and DRG filopodia is the higher presence of MTs inside hippocampal filopodia. By immunostaining we observed that in hippocampal GCs more MTs extend into the P domain of GCs and enter the proximal part of filopodia (Figure 5). MTs are a major cytoskeletal component during neuronal development and play an active role in neurite growth and axon specification [28,34,35]. Individual MTs inside filopodia or within the P domain of the GC undergo cycles of growth and catastrophe, “dynamic instability”, which enables MTs to quickly remodel their organization and selectively grow in response to extracellular signals [36] and influence membrane protrusion [36,37]. If a filament behaves as a homogeneous elastic rod the magnitude of buckling forces is proportional to the flexural rigidity of the filament itself [38]. The mean flexural rigidity of MTs is 2.2 x 10^-23^ Nm^2^ which is almost 1000 times larger than that of actin filaments equal to 7.3 10^-26^ Nm^2^ [32]. All these considerations suggest that the presence of MTs inside filopodia of hippocampal GC is the main reason why hippocampal filopodia exert a larger force than DRG filopodia.

### Hippocampal and DRG lamellipodia

Our experimental data indicate that the maximal measured force depends on the contact area between the bead and the lamellipodium leading edge [19,22]. In these experiments, the bead diameter was 1 µm and the area in contact with the silica bead, A_c_, obtained from videomicrographs, varied from less than 0.1 up to 1.5 µm^2^. Therefore, we expect the contact area between a bead and a larger lamellipodium to be higher, on average, than that with a smaller lamellipodium providing one reason why DRG lamellipodia exert a larger force. Another possibility is that larger lamellipodia are more rigid than smaller lamellipodia, as a consequence of a stronger structural stability caused by a more extensive crosslinking of connecting proteins [39], such as myosins and other regulatory proteins. Our results show that the mean size of DRG GCs (P10-P12) is almost 10 times larger than hippocampal GCs (Figure 6) and the corresponding DRG lamellipodia are much larger. In both types of neurons the maximum exerted force by lamellipodia increases when the size of GC is larger (Figure S2 in Information S1), showing that the lamellipodium area affects the exerted force. Interestingly, the mean size of a hippocampal GC obtained from P1-P2 and P10-P12 rats remains constant, which suggests that the maximal exerted force by lamellipodia in hippocampal neurons must not change, in agreement with our experimental data. Videoimaging of DRG neurons after 3-12 hours of culture shows that vigorous lamellipodia emerge directly from the soma and protrude and collapse continuously undergoing three dimensional motions and that their leading edges are able to lift up by 3-5 µm and to exert a larger vertical force than hippocampal lamellipodia (Table 1).

The results here presented are in agreement with recent studies [40] showing that the DRG GCs exert a larger traction force than hippocampal GCs. This difference was attributed to the larger density of paxillin, an adhesion molecule, significantly higher in DRG than in hippocampal GCs [40], suggesting that the difference in force generation could be due to the stronger adhesion of the GC to the substrate. We used optical tweezers providing a better time resolution and accuracy allowing a precise determination of the force exerted by filopodia and lamellipodia.

### Functional role

Our results indicate that the different morphology of GCs, which can vary widely among neuronal cell types, affect their motility and force generation. Morphological differences in CNS and PNS neurons are probably due to their function: neurons from the CNS develop, migrate and navigate usually over glial cells, which from a mechanical point of view is a softer substrate [41,42] but the environment where PNS neurons navigate is different [41,43]. We suggest that the location where a neuron develops, migrates and navigates determines the properties of the force which filopodia and lamellipodia must develop. DRG GCs by using their strong lamellipodia travel long distances before reaching their final target. Hippocampal GCs move among a multitude of other neurons and glial cells and their filopodia must explore a crowded environment and find their final destination. Therefore, perhaps, it is convenient to have smaller GCs but with stronger filopodia. The mechanical properties of the environment and the functional role of the exploring GC require lamellipodia and filopodia to exert very precise forces which are different in the CNS and in the PNS.

## Supporting Information

Information S1 **This file provides additional information about different fixation protocols used for immunostaining of actin and tubulin (Figure S1) and a figure which represent the relation between area of GC and maximum exerted force (Figure S2).**
(PDF)

## References

[1] Ghashghaei HT, Lai C, Anton ES (2007) Neuronal migration in the adult brain: are we there yet? Nat Rev Neurosci 8: 141–151. doi:10.1038/nrn2074. PubMed: 17237805. 17237805 10.1038/nrn2074

[2] Solecki DJ, Govek EE, Hatten ME (2006) mPar6 alpha controls neuronal migration. J Neurosci: 26: 10624–10625. doi:10.1523/JNEUROSCI.4060-06.2006. PubMed: 17050699. 17050699 10.1523/JNEUROSCI.4060-06.2006 PMC6674730

[3] Bray D, Thomas C, Shaw G (1978) Growth cone formation in cultures of sensory neurons. Proc Natl Acad Sci U S A 75: 5226–5229. doi:10.1073/pnas.75.10.5226. PubMed: 283427. 283427 10.1073/pnas.75.10.5226 PMC336299

[4] Goodman CS (1996) Mechanisms and molecules that control growth cone guidance. Annu Rev Neurosci 19: 341–377. doi:10.1146/annurev.ne.19.030196.002013. PubMed: 8833447. 8833447 10.1146/annurev.ne.19.030196.002013

[5] Song H, Poo M (2001) The cell biology of neuronal navigation. Nat Cell Biol 3: E81–E88. doi:10.1038/35060164. PubMed: 11231595. 11231595 10.1038/35060164

[6] Mongiu AK, Weitzke EL, Chaga OY, Borisy GG (2007) Kinetic-structural analysis of neuronal growth cone veil motility. J Cell Sci 120: 1113–1125. doi:10.1242/jcs.03384. PubMed: 17327278. 17327278 10.1242/jcs.03384

[7] Suter DM, Forscher P (2000) Substrate – Cytoskeletal Coupling as a Mechanism for the Regulation of Growth Cone Motility and Guidance. J Neurobiol 44: 97–113. doi:10.1002/1097-4695(200008)44:2. PubMed: 10934315. 10934315

[8] Pollard TD, Borisy GG (2003) Cellular motility driven by assembly and disassembly of actin filaments. Cell 112: 453–465. doi:10.1016/S0092-8674(03)00120-X. PubMed: 12600310. 12600310 10.1016/s0092-8674(03)00120-x

[9] Mogilner A, Oster G (1996) Cell motility driven by actin polymerization. Biophys J 71: 3030–3045. doi:10.1016/S0006-3495(96)79496-1. PubMed: 8968574. 8968574 10.1016/S0006-3495(96)79496-1 PMC1233792

[10] Pak CW, Flynn KC, Bamburg JR (2008) Actin-binding proteins take the reins in growth cones. Nat Rev Neurosci 9: 136–147. doi:10.1038/nrn2236. PubMed: 18209731. 18209731 10.1038/nrn2236

[11] Howard J (2001) Mechanics of Motor Proteins and the Cytoskeleton. Sunderland, MA: Sinauer.

[12] Raucher D, Sheetz MP (2000) Cell spreading and lamellipodial extension rate is regulated by membrane tension. J Cell Biol 148: 127–136. doi:10.1083/jcb.148.1.127. PubMed: 10629223. 10629223 10.1083/jcb.148.1.127 PMC2156205

[13] Carlsson AE (2001) Growth of branched actin networks against obstacles. Biophys J 81: 1907–1923. doi:10.1016/S0006-3495(01)75842-0. PubMed: 11566765. 11566765 10.1016/S0006-3495(01)75842-0 PMC1301666

[14] Carlsson AE (2003) Growth velocities of branched actin networks. Biophys J 84: 2907–2918. doi:10.1016/S0006-3495(03)70018-6. PubMed: 12719223. 12719223 10.1016/S0006-3495(03)70018-6 PMC1302854

[15] Mogilner A, Oster G (2003) Force generation by actin polymerization II: the elastic ratchet and tethered filaments. Biophys J 84: 1591–1605. doi:10.1016/S0006-3495(03)74969-8. PubMed: 12609863. 12609863 10.1016/S0006-3495(03)74969-8 PMC1302730

[16] Mogilner A (2009) Mathematics of cell motility: have we got its number? J Math Biol 58: 105–134. doi:10.1007/s00285-008-0182-2. PubMed: 18461331. 18461331 10.1007/s00285-008-0182-2 PMC2862828

[17] Neuman KC, Block SM (2004) Optical trapping. Rev Sci Instrum 75: 2787–2809. doi:10.1063/1.1785844. PubMed: 16878180. 16878180 10.1063/1.1785844 PMC1523313

[18] Bustamante C, Macosko JC, Wuite GJ (2000) Grabbing the cat by the tail: manipulating molecules one by one. Nat Rev Mol Cell Biol 1: 130–136. doi:10.1038/35040072. PubMed: 11253365. 11253365 10.1038/35040072

[19] Shahapure R, Difato F, Laio A, Bisson G, Ercolini E et al. (2010) Force generation in lamellipodia is a probabilistic process with fast growth and retraction events. Biophys J 98: 979–988. doi:10.1016/j.bpj.2009.11.041. PubMed: 20303855. 20303855 10.1016/j.bpj.2009.11.041 PMC2849058

[20] Tosney KW, Landmesser LT (1985) Growth Cone Morphology and Trajectory in the Lumbosacral Region of the Chick Embryo. J Neurosci 5: 2345–2358. PubMed: 4032000. 4032000 10.1523/JNEUROSCI.05-09-02345.1985 PMC6565320

[21] Melli G, Höke A (2009) Dorsal Root Ganglia Sensory Neuronal Cultures: a tool for drug discovery for peripheral neuropathies. Expert Opin Drugs Discov 4: 1035–1045. doi:10.1517/17460440903266829. PubMed: 20657751. 10.1517/17460440903266829 PMC2908326 20657751

[22] Amin L, Ercolini E, Shahapure R, Bisson G, Torre V (2011) The elementary events underlying force generation in neuronal lamellipodia. Scientific Rep 1: 153. doi:10.1038/srep00153. PubMed: 22355669. 10.1038/srep00153 PMC3240973 22355669

[23] Amin L, Ercolini E, Shahapure R, Migliorini E, Torre V (2012) The role of membrane stiffness and actin turnover on the force exerted by DRG lamellipodia. Biophys J 102: 2451–2460. doi:10.1016/j.bpj.2012.04.036. PubMed: 22713560. 22713560 10.1016/j.bpj.2012.04.036 PMC3368133

[24] Ruaro ME, Bonifazi P, Torre V (2005) Toward the neurocomputer: image processing and pattern recognition with neuronal cultures. IEEE Trans Bio Med Eng 52: 371–383. doi:10.1109/TBME.2004.842975. PubMed: 15759567. 10.1109/TBME.2004.842975 15759567

[25] Cojoc D, Difato F, Ferrari E, Shahapure RB, Laishram J et al. (2007) Properties of the force exerted by filopodia and lamellipodia and the involvement of cytoskeletal components. PLOS ONE 2: e1072. doi:10.1371/journal.pone.0001072. PubMed: 17957254. 17957254 10.1371/journal.pone.0001072 PMC2034605

[26] Kress H, Stelzer EH, Griffiths G, Rohrbach A (2005) Control of relative radiation pressure in optical traps: Application to phagocytic membrane binding studies. Phys Rev E 71: 061927. doi:10.1103/PhysRevE.71.061927. PubMed: 16089785. 10.1103/PhysRevE.71.061927 16089785

[27] Pool M, Thiemann J, Bar-Or A, Fournier AE (2008) NeuriteTracer: a novel ImageJ plugin for automated quantification of neurite outgrowth. J Neurosci Methods 168: 134–139. doi:10.1016/j.jneumeth.2007.08.029. PubMed: 17936365. 17936365 10.1016/j.jneumeth.2007.08.029

[28] Vitriol EA, Zheng JQ (2012) Growth cone travel in space and time: the cellular ensemble of cytoskeleton, adhesion, and membrane. Neuron 73: 1068–1081. doi:10.1016/j.neuron.2012.03.005. PubMed: 22445336. 22445336 10.1016/j.neuron.2012.03.005 PMC3319373

[29] Xiong Y, Lee AC, Suter DM, Lee GU (2009) Topography and nanomechanics of live neuronal growth cones analyzed by atomic force microscopy. Biophys J 96: 5060–5072. doi:10.1016/j.bpj.2009.03.032. PubMed: 19527666. 19527666 10.1016/j.bpj.2009.03.032 PMC2712036

[30] Dent EW, Gertler FB (2003) Cytoskeletal dynamics and transport in growth cone motility and axon guidance. Neuron 40: 209–227. doi:10.1016/S0896-6273(03)00633-0. PubMed: 14556705. 14556705 10.1016/s0896-6273(03)00633-0

[31] Laishram J, Kondra S, Avossa D, Migliorini E, Lazzarino M et al. (2009) A morphological analysis of growth cones of DRG neurons combining atomic force and confocal microscopy. J Struct Biol 168: 366–377. doi:10.1016/j.jsb.2009.09.005. PubMed: 19747551. 19747551 10.1016/j.jsb.2009.09.005

[32] Gittes F, Mickey B, Nettleton J, Howard J (1993) Flexural rigidity of microtubules and actin filaments measured from thermal fluctuations in shape. J Cell Biol 120: 923–934. doi:10.1083/jcb.120.4.923. PubMed: 8432732. 8432732 10.1083/jcb.120.4.923 PMC2200075

[33] Dotti G, Sullivan CA, Banker GA (1988) The Establishment of Polarity by Hippocampal Neurons in Culture. J Neurosci 8(4): 1454–1468. PubMed: 3282038. 3282038 10.1523/JNEUROSCI.08-04-01454.1988 PMC6569279

[34] Schaefer AW, Kabir N, Forscher P, Haven N (2002) Filopodia and actin arcs guide the assembly and transport of two populations of microtubules with unique dynamic parameters in neuronal growth cones. J Cell Biol, 158: 139–152. doi:10.1083/jcb.200203038. PubMed: 12105186. 12105186 10.1083/jcb.200203038 PMC2173029

[35] Dent EW, Gupton SL, Gertler FB (2011) The growth cone cytoskeleton in axon outgrowth and guidance. Cold Spring Harbor Perspectives Biol 3: ([MedlinePgn:]) doi:10.1101/cshperspect.a001800. PubMed: 21106647. 10.1101/cshperspect.a001800 PMC3039926 21106647

[36] Buck KB, Zheng JQ (2002) Growth Cone Turning Induced by Direct Local Modification of Microtubule Dynamics. J Neurosci 22: 9358–9367. PubMed: 12417661. 12417661 10.1523/JNEUROSCI.22-21-09358.2002 PMC6758015

[37] Mack TGA, Koester MP, Pollerberg GE (2000) The Microtubule-Associated Protein MAP1B Is Involved in Local Stabilization of Turning Growth Cones. Mol Cell Neurosci 65: 51–65. doi:10.1006/mcne.1999.0802. PubMed: 10662505. 10.1006/mcne.1999.0802 10662505

[38] Dogterom M, Yurke B (2012) Measurement of the Force-Velocity Relation for Growing Microtubules Measurement of the Force-Velocity Relation for Growing Microtubules. Science 856. doi:10.1126/science.278.5339.856. 10.1126/science.278.5339.856 9346483

[39] Laurent M, Kasas S, Yersin A, Scha TE, Dietler G et al. (2005) Gradient of Rigidity in the Lamellipodia of Migrating Cells Revealed by Atomic Force Microscopy. Biophys J 89: 667–675. doi:10.1529/biophysj.104.052316. PubMed: 15849253. 15849253 10.1529/biophysj.104.052316 PMC1366565

[40] Koch D, Rosoff WJ, Jiang J, Geller HM, Urbach JS (2012) Strength in the periphery: growth cone biomechanics and substrate rigidity response in peripheral and central nervous system neurons. Biophys J 102: 452–460. doi:10.1016/j.bpj.2011.12.025. PubMed: 22325267. 22325267 10.1016/j.bpj.2011.12.025 PMC3274825

[41] Moore SW, Roca-Cusachs P, Sheetz MP (2010) Stretchy proteins on stretchy substrates: the important elements of integrin-mediated rigidity sensing. Dev Cell 19: 194–206. doi:10.1016/j.devcel.2010.07.018. PubMed: 20708583. 20708583 10.1016/j.devcel.2010.07.018 PMC5319208

[42] Christ AF, Franze K, Gautier H, Moshayedi P, Fawcett J et al. (2010) Mechanical difference between white and gray matter in the rat cerebellum measured by scanning force microscopy. J Biomech 43: 2986–2992. doi:10.1016/j.jbiomech.2010.07.002. PubMed: 20656292. 20656292 10.1016/j.jbiomech.2010.07.002

[43] Franze K, Guck J (2010) The biophysics of neuronal growth. Rep Prog Phys 73: 094601. doi:10.1088/0034-4885/73/9/094601.

