# Supplementary material for: Comparison of the Force Exerted by Hippocampal and DRG Growth Cones
Source: PLoS One. 2013 Aug 21;8(8):e73025. doi: 10.1371/journal.pone.0073025 (PMC3749134; doi:10.1371/journal.pone.0073025)
Supplement: Information S1 — This file provides additional information about different fixation protocols used for immunostaining of actin and tubulin (Figure S1) and a figure which represent the relation between area of GC and maximum exerted force (Figure S2). (PDF) [file pone.0073025.s001.pdf]

Comparison of the Force Exerted by Hippocampal and DRG Growth Cones

Ladan Amin,[1] Erika Ercolini,[1,2] Jelena Ban[1] and Vincent Torre[1,3*]

Supplementary Information S1

Immunostaining of actin and tubulin. Since microtubules are sensitive to fixation procedure and in order to check the reliability of our results several fixation protocols were tested for microtubules.

Method 1. 4% paraformaldehyde (PFA) 20 min room temperature (RT) used in this study as reference
Method 2. 4% PFA 20 min RT with 5 min permeabilization with ice-cold methanol
Method 3. 100% ice-cold methanol 15 min -20°C
Method 4. 100% ice-cold methanol 10 min -20°C followed by 100% cold acetone 5 min -20°C
Method 5. 4% PFA -0.25% glutaraldehyde 20 min RT

The cells were fixed 24 h after plating and stained for tubulin (TUJ1 Antibody) and actin (Phalloidin-488 Alexa). However, in all methods where methanol were used (2,3 and 4) actin staining was significantly decreased since methanol fixation destroys the phalloidin-binding site on actin [1]. Actin staining is fundamental when GCs are imaged since F-actin is the most abundant protein in the GCs and it is mainly located in peripheral domain and filopodia where microtubules are less abundant. Another disadvantage of methanol fixation is that distorts the GC three-dimensional structure which was fundamental for the morphological analysis shown in Fig. 6. For this reason we used crosslinking fixatives such as PFA. We tested also glutaraldehyde (method 5) that however preserves less well the actin microfilaments compared to aldehyde fixatives.

As shown in the figure below (Figure S1), microtubule staining were strong and convincing when the protocols 2,3,4 were performed but these fixation protocols destroyed the GC 3D structure; in fact, most of the GCs were flat and merged images show almost total overlapping of actin and tubulin staining, while only protocol 1 and 5 provided optimal staining both for actin and microtubules. Therefore we can conclude that by using different fixation protocols we confirmed

that in hippocampal GCs microtubules extend into the periphery domain and even inside filopodia (Figure S1).

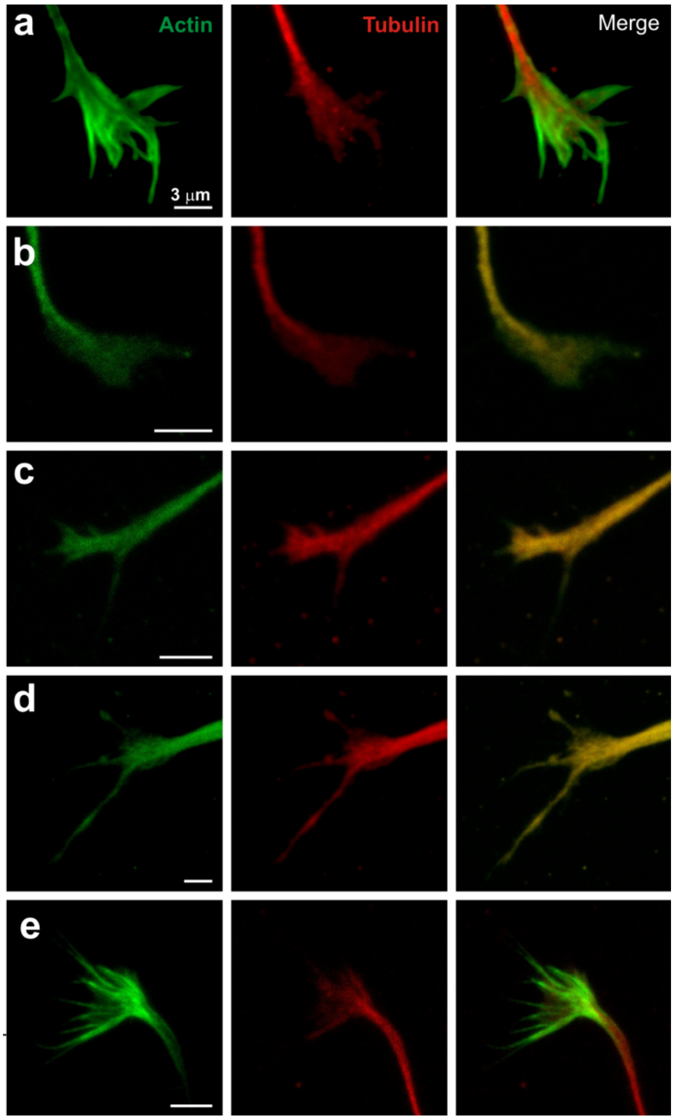

Figure S1. Different fixation protocols for immunostaining of actin and tubulin. (*a*) From left to right: confocal fluorescence images of a hippocampal GC stained for actin, tubulin and merge of the two staining where cells were fixed using method 1. (*b*-*e*) As in (a) but cells were fixed using method 2,3,4 and 5, respectively.

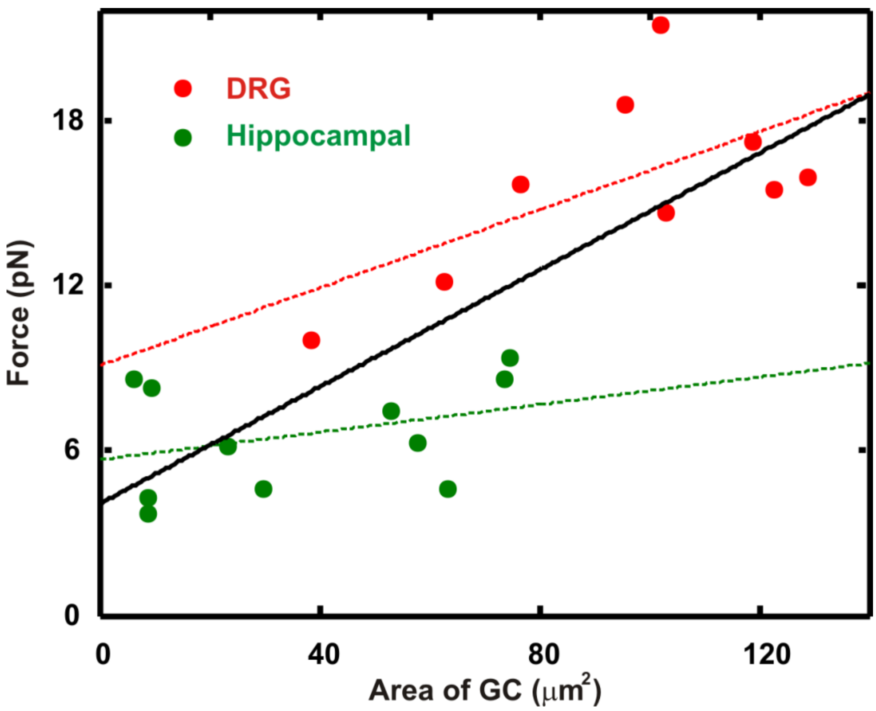

Figure S2. Relation between area of GC and maximum exerted force by GC. By increasing the size of GC the maximum exerted force by its lamellipodia increases for both hippocampal (green symbols) and DRG (red symbols) GCs. Lines are linear fits for experimental data collected from hippocampal (dotted green line) DRG (dotted red line) GCs and black line represent the linear fit for all samples. Correlation coefficients is equal to 0.77 (P value $<10^{-3}$).

REFERENCES

1. Haugland RP (1998) Handbook of fluorescent probes and research chemicals.
